# Foods, Drugs, Instruments, &c.

**Published:** 1890-08-30

**Authors:** 


### A MEDICAL MUSEUM.

### FOOD, DRUGS, INSTRUMENTS, &c.

Every year the British Medical Association meets at one or other of our large centres of population; and every year the most energetic and enterprising firms who specially prepare foods, drugs, and instruments for medical purposes, follow the Association about with a travelling museum or exhibition of the finest specimens of the various articles which they wish particularly to urge upon the critical attention of the doctors. The exhibition has now become thoroughly well known both to manufacturers and medical men; and there is no better way either of making known the virtues of a new preparation, or of extending the reputation of an old one than that of showing it off to advantage at the annual exhibition of the British Medical Association. This year the meeting of the Association was held at Birmingham; and to many minds the wonderful variety of natural and artificial pathological specimens, and the beautiful display of instruments, delicately-prepared drugs, and appetising and nutritious foods was the most interesting of all that was to be seen or heard on the occasion. It would be of great service to hospitals and medical practitioners if we could give a detailed description of many of the articles exhibited; but the limits of space forbid anything more than a cursory glance at some of the more important preparations of the best known exhibitors.

Messrs. Ingram and Royle showed a great variety of improved mineral waters, among which those of the Vichy State springs occupied a prominent place. It is not necessary to say anything in praise of the Vichy waters. As natural mineral waters, highly alkalised, they have probably no rival. The single fact that during the year 1888 no fewer than 6,828,278 bottles were sold is proof positive that, both in the medical profession and among the general public, there is a solidly-established confidence in the sterling value of the waters of Vichy. The same firm exhibited the Carlsbad waters and also the Sprudel salts in the form of lozenges, which are taken for acidity of the stomach, either alone or with the Carlsbad water.

The Liquor Carnis Company, of 50, High Holborn, showed specimens of their uncooked meat-juice or muscle plasma. We have already expressed a very decided judgment as to the value of this new food. In the wasting diseases of infants and other invalids, it is probably destined to become one of the chief medicinal and food agents used in practice.

The Bouillon Fleet Company (Warner Road, Camberwell) exhibited samples of Bouillon Fleet and Bouillon Fleet Cartridges. Bouillon, it is stated, is prepared from fresh beef only, and, being a peptone of beef, is very easy of digestion. One cartridge will make a small cup of well-tasted soup, and is very easily prepared.

Messrs. Brand and Company, whose reputation is thoroughly established, had among their exhibits Invalid Meat Biscuits, Extractum Carnis, and Beef Bouillon. Those preparations are among the best of their kind, and are sold at very moderate prices.

Messrs. Carnrick and Company's preparations, including Beef Peptonoids, digested and dissolved in sherry; Liquid Peptonoids, with coca, iron, and wine; Soluble Food, consisting of evaporated milk, partly peptonised and completely steralised, combined with dextrine and milk sugar, dried and mixed for use as a fine powder, were shown by the Maltine Manufacturing Company. These preparations are well and favourably known in the medical profession.

Messrs. Denaeyer and Company (118, Bishopsgate Street) exhibited samples of their Peptonate of Iron, containing 20 per cent. of a pure peptone and 2 per cent. of Ferric Hydrate. The taste of the iron is almost imperceptible, and the combination may prove very valuable where other tonics cannot be taken. The Liquid Sterilised Peptone of Meat prepared by this company is said to contain as large a percentage of proteid matter as lean beef, and is thoroughly digested before use. It has a fine flavour and an appetising appearance. It is also prepared as a powder, which will dissolve in wine, soup, or beef-tea.

The Liebig's Wine Company were to the fore with "Vivo," a pure beef juice, which claims to combine all the tonic and nutritive properties of meat in an agreeable and highly-concentrated form. They also showed Vin Pregno, a beef wine made with Liebig's Extract. The Liebig's Wine Company is at Highfield Street, Liverpool.

Mr. J. Mellin, of the Marlborough Works, Peckham, presented samples of Mellin's Food for Infants, and also Mellin's Lacto-glycose. Mellin's infant food is widely and favourably known as one of the best and safest of its class. The firm exhibited a new product known as Mellin's Food Biscuits. They contain equal parts of Mellin's food and fine wheat flour, and form a pleasant and palatable solid food for children and others to whom an exclusively liquid diet is not suited. A good many other firms exhibited valuable articles of diet, to which we may refer at some future time.

The drugs and pharmaceutical preparations, it is needless to say, were not only "elegant," but of a high order of merit. Pharmacy, indeed, has almost outstripped medicine in the race for improvement. It is not possible as yet to deprive all drugs of their "nasty" character; but a variety of most ingenious devices is resorted to whereby the "nastiness," if not got rid of, is at least reduced to a minimum. Some of the best known and most trusted of British and American pharmacists exhibited samples of their new, and, generally, admirable preparations.

Messrs. Allen and Hanbury, of Plough Court, Lombard Street, made a beautiful display of some of their new preparations. Among these may be mentioned Bynin, a fluid malt extract; Bynol, a combination of malt with cod liver oil; Byno-pepsine; Byno-pancreatine; Byno-hypophosphites, and other tonic malt compounds. These preparations are very useful as tonics, and are stated to keep well. The firm also showed their well-known perfected Cod Liver Oil and Tasteless Castor Oil. Of their recent preparations they exhibited compressed drugs, granular effervescent medicaments, Cascara Sagrada lozenges and capsules, Salol, Exalgine, and many others. This well-known firm has an established reputation for every kind of pharmaceutical excellence; and it is needless to say that their various preparations were of the highest class.

Messrs. Corbyn and Stacey, of the Poultry, and New Bond Street, exhibited a large collection of new drugs and pharmaceutical preparations. Amongst the articles recently introduced may be mentioned the following: Somnal, a new hypnotic; Sulphaminol, an antiseptic powder, used as a substitute for iodoform; Aristol, also an antiseptic and iodoform substitute; Oresin, a new tonic and stomachic, and many other new and valuable remedies. The exhibits of this firm well illustrated the advance of pharmacy during the year, and can hardly be too highly praised.

Messrs. W. H. Schieffelin and Company, of New York, who were represented by Mr. W. E. Sacker, ot Fenchurch Street, exhibited a considerable variety of their beautiful gelatine coated Granules and Pills. If it be possible to make a pill positively pleasant, Messrs. Schieffelin may claim the honour of having accomplished this desirable object. In many instances they have discarded the old spheroidal shapes, and have adopted instead ovate and other more alluring forms. The variously coloured coatings in which many of their medicaments are encased leave nothing to be desired in the way of beauty and attractiveness. It is hardly too much to say of the Messrs. Schieffelin that they have revolutionised the pill world.

Messrs. G. and G. Stern, of Gray's Inn Road, showed in various forms their Pumiline Essences. These include a Pumiline Linament, a Pumiline Ointment, and a Pumiline Emulsion. The Emulsion is made by suspending the Pumiline Essence in distilled water with a little soda and glycerine. These preparations are often exceedingly useful in throat and chest affections. A new remedy, called Pepsalia, which consists of several digestive ferments with common salt and free hydrochloric acid was exhibited and distributed about the rooms by the same firm. It resembles in taste and appearance fine table salt, and is intended to be substituted for it at the table. This novel preparation is stated to possess great activity as a digestive agent, and some interesting experiments which were made in the presence of visitors with boiled white of egg, amply justified this contention.

Messrs. Southall Brothers and Barclay, of Birmingham, whose speciality is the presentation of drugs in the crude form, showing the varying proportion of the active principles in different samples of the same denomination, had some interesting exhibits, among these were several specimens of Indian drugs, collected by Mr. David Hooper, F.C.S., Quinologist to the Madras Government. These samples of crude drugs showed well how tinctures and other medicaments may vary in strength and efficacy, though quite honestly prepared from the substances of which they are the representatives. As might have been expected, this was particularly well illustrated in the case of cinchona. Several samples of tincture cinchonæ were shown, and the variation in strength was as much as 170 per cent. Southall's concentrated tinctures, which formed a group by themselves, are four times the strength of the ordinary standard tinctures, and are all assayed. A great saving is effected in hospitals and extensive general practices by the use of such tinctures. Messrs. Southall also showed their well-known sanitary towels and sheets, and a variety of nurses, students, and practitioners' requisites, including a District Nurse's Basket, Binaural Stethoscopes, midwives and nurses' fitted bags, Southall's urometer and urine testing cabinets, hot-air baths, bronchitis kettles, &c. The display of all these modern indispensables illustrates admirably the progress of that important part of medical and surgical treatment which goes under the general description of sick-room management.

We have left ourselves but little space for the noticing of surgical requisites, yet the antiseptic dressings, the bandages, the plasters, the ointments, and the instruments of every conceivable and inconceivable form, size, and use were innumerable. It is evident that the specialist is abroad in every department of medicine and surgery. How otherwise would even the names, much less the uses, of the various instruments and appliances ever be known?

Messrs. Philip Harris and Company, of Birmingham, exhibited Polariscopes, chemical balances, medical batteries, microscopes, antidote bags, Ehrendorfer's pencils, surgical instrument cases, and everything else that the most exacting scientist could desire. All these various instruments and appliances were conceived with wonderful ingenuity, and made and finished in the highest perfection of handiwork.

Messrs. Johnson and Johnson, of New York, covered a table with antiseptic dressings, medicinal plasters, and other specialties. Their Cantharidian Plaster, which they call "Canthos," is alleged to be the best blister in the world. How happy must certain patients be when they hear of this advance in blisters! In the United States this particular plaster is said to have far out-distanced every rival. Among other advantages that it claims are these: That it acts in about half the time of the ordinary vesicant; that it does not decompose with keeping; that it does not adhere too closely, but yet closely enough; that it is easily applied, requiring no oil or other preparation; and that it does not produce unbearable irritation. What happy people Messrs. Johnson and Johnson, of New York, ought to be in having provided the profession with a blister that is not unbearable!

After so much physic and so many medical appliances, a cup of tea would be not unwelcome; and the Universal Digestive Tea Company, of Market Street, Manchester, were at Birmingham in force, tempting the doctors to taste and try. The Universal Digestive Tea is prepared upon the principle of having its tannin neutralised. It is said to be particularly suited to nursing mothers, dyspeptics, and invalids generally. If it be the case that the tannin is rendered inactive, it is probable that the tea will gain much in digestibility. At any rate it is worth a trial, and the proprietors showed some confidence in their article when they submitted it to the critical investigation of the members of the British Medical Association.

